# Effects of age and nutritional state on the expression of gustatory receptors in the honeybee (*Apis mellifera*)

**DOI:** 10.1371/journal.pone.0175158

**Published:** 2017-04-12

**Authors:** Nicola K. Simcock, Luisa A. Wakeling, Dianne Ford, Geraldine A. Wright

**Affiliations:** 1 Institute of Neuroscience, Newcastle University, Newcastle upon Tyne, United Kingdom; 2 School of Dental Sciences, Newcastle University, Newcastle upon Tyne, United Kingdom; 3 Institute for Cell and Molecular Biosciences, Medical School, Newcastle University, Newcastle upon Tyne, United Kingdom; Universidade de Sao Paulo Faculdade de Filosofia Ciencias e Letras de Ribeirao Preto, BRAZIL

## Abstract

Gustatory receptors (Grs) expressed in insect taste neurons signal the presence of carbohydrates, sugar alcohols, CO_2_, bitter compounds and oviposition stimulants. The honeybee (*Apis mellifera*) has one of the smallest Gr gene sets (12 Gr genes) of any insect whose genome has been sequenced. Honeybees live in eusocial colonies with a division of labour and perform age-dependent behavioural tasks, primarily food collection. Here, we used RT-qPCR to quantify Gr mRNA in honeybees at two ages (newly-emerged and foraging-age adults) to examine the relationship between age-related physiology and expression of Gr genes. We measured the Gr mRNAs in the taste organs and also the brain and gut. The mRNA of all Gr genes was detected in all tissues analysed but showed plasticity in relative expression across tissues and in relation to age. Overall, Gr gene expression was higher in the taste organs than in the internal tissues but did not show an overall age-dependent difference. In contrast Gr gene expression in brain was generally higher in foragers, which may indicate greater reliance on internal nutrient sensing. Expression of the candidate sugar receptors AmGr1, AmGr2 and AmGr3 in forager brain was affected by the types of sugars bees fed on. The levels of expression in the brain were greater for AmGr1 but lower for AmGr2 and AmGr3 when bees were fed with glucose and fructose compared with sucrose. Additionally, AmGr3 mRNA was increased in starved bees compared to bees provided ad libitum sucrose. Thus, expression of these Grs in forager brain reflects both the satiety state of the bee (AmGr3) and the type of sugar on which the bee has fed.

## Introduction

The chemical sense of gustation enables food identification and toxin avoidance. In insects, the pre-ingestive assessment of tastants relies on a diverse set of gustatory receptors (Grs) expressed across chemosensory tissues such as the labial palps, antennae and tarsi. These receptors are located in the membranes of gustatory receptor neurons (GRNs) housed in hair-like *sensilla chaetica* and *sensilla basiconica* [1–3]. Insect Grs have a membrane topology that is an inverted version of the G-protein coupled receptors (GPCRs) of mammals, with an intracellular N-terminus and extra cellular C-terminus [4, 5]. Studies using G-protein signalling inhibitors, gamma subunit suppression, null mutants and RNA-interference (RNAi) indicate that both G-protein-dependent and -independent signal transduction pathways mediate gustatory coding [6–9], but the exact function of insect Grs is still unclear. Thus, the insect gustatory system is highly complex and knowledge concerning its function and regulation is currently lacking.

All animals rely on their chemical senses to assess the dynamic external environment. Gustation in particular allows animals to identify potential food sources, aiding efficient selection of nutritious foods and detection and aversion by noxious chemicals, often represented by a bitter taste. For many insect species, in-depth functional assessment of Grs expressed in the canonical chemosensory tissues, which we refer to here as ‘external’ Grs, is ongoing. However, in an increasing number of insect species gustatory receptor gene expression has been observed in a number of ‘internal’ non-canonical tissues, including the brain and gut [9–11] and is assumed to be associated with post-ingestive nutrient sensing. The most convincing evidence to date concerns the narrowly tuned *Drosophila* fructose receptor DmGr43a. In addition to its presence in classic gustatory appendages, DmGr43a was detected in a small number of brain neurons[11]. While glucose and trehalose are generally the most abundant sugars in Drosophila haemolymph (~9–13 μg/mg) and remain relatively stable regardless of feeding, fructose is found at lower concentrations (~0.07 μg/mg) but increases up to 10-fold following a meal [11]. Elevated fructose levels in the haemolymph were shown to activate the DmGr43a-expressing brain neurons and were strongly correlated with the continuation or cessation of feeding in a satiation-dependent manner [11].

To achieve nutritional homeostasis, appropriate responses to external stimuli must be coupled with the accurate assessment of internal status. While we know of some proteins and regulatory pathways associated with feeding regulation, for example insulin-like peptides (ILPs) and adipokinetic hormones (AKH) [12–15], it is becoming apparent that this important mechanism may additionally involve gustatory receptors.

In the assessment of Gr function, the honeybee offers a unique advantage by possessing one of the smallest Gr gene repertoires of any insect species annotated to date (12 Gr genes) [16–18]. The Gr1 and Gr2 genes are likely to encode sugar receptors [16, 19]. A high level of conservation amongst the Dm43a-like receptors additionally suggests that the Gr3 gene will likely play a role in fructose detection [9, 11, 20]. Little is known about the function of the other Gr genes—particularly Gr12, which has been discovered only recently [18]. There is weak sequence similarity between Gr4 and Gr5 and the *Drosophila* bitter receptor complex DmGr28a-e [16], Gr11 is thought to be a pseudogene [17]. All remaining *Apis* Grs may potentially represent a species-specific lineage. Similarly, the recently published *Bombus terrestris* genome includes a number of additional duplications among these unknown genes[18]. While ligand specificity is still unknown the authors highlight that the *Bombus* expansion may play a role in the ‘more diverse nest-building habits’ of bumblebees compared to honeybees [18]. As we lack total functional analysis for any honeybee Gr, in the current work we refer to the genes as ‘candidate’ receptor genes for the function we speculate they have, such as AmGr1 and AmGr2 as candidate sugar receptors.

As adult worker honeybees age, they progress through a series of behavioural castes including nursing, wax building, guarding, and lastly, foraging [21]. The transition from the time of eclosion to foraging is accompanied by a number of metabolic, physiological and behavioural changes [22–26]. Young adult worker honeybees consume pollen—which is mainly made of protein and fats—to produce glandular secretions to feed the larvae, the queen, and other adult workers [27, 28]. In contrast, adult forager honeybees subsist on a diet that is primarily carbohydrate [21, 29]. Gustatory sensitivity increases following the transition from in-hive tasks to foraging, resulting from an increase in juvenile hormone (JH) and a decrease in vitellogenin protein titre [30,31].

While Grs are important for the detection of nutrients regardless of age, foraging-age workers are more likely to encounter toxins from plants as they forage [32] and less likely to consume pollen [33]. Interestingly, the hypopharyngeal and mandibular glands of forager bees are enriched in transcripts for detoxification enzymes, antimicrobial peptides and immune responses, compared to nurse bees [34] Additionally, due to the physiological differences between honeybee age castes, there are also differences between nutrient requirement and regulation.

Both long and short term changes in gustatory sensitivity, including physical changes in sensilla number, have been previously reported in insects [35–37] but few have examined how Gr expression changes as a function of age in insects, or indeed nutrition. In the present study we use quantitative reverse transcription PCR (RT-qPCR) to measure expression of the 10 Gr genes discovered initially [16] throughout the tissues of newly emerged and forager honeybees. Additionally, we explored the effect of diet-restriction and carbohydrate consumption on Gr expression after observing internal expression and apparent plasticity. We chose to focus on a sugar-only diet, firstly because the honeybee diet primarily consists of sugars and we knew both forager and nurse bees would survive throughout the experiment. Secondly, we are most confident that honeybees possess sugar receptors as suggested by strong homology and an experimental study [16, 19]. While honeybees are unlikely to collect individual sugars in nature, some nectar rewards are dominated by sucrose and others by a mixture of glucose and fructose [38] however, offering one sugar alone allowed us to investigate whether receptor gene expression was influenced by individual sugars.

Improvement in genome annotation since Robertson and Wanner’s [16] study of 2006 now predicts splice variants for Gr1, Gr6 and Gr10 bringing the total number of putative honeybee Grs to 18 [39]. However, molecular studies investigating the existence of these variants are lacking. For the purpose of this study we therefore investigated expression of the 10 original Gr genes in internal tissues (brain and gut) and also in the sensory organs (antennae, galea, labial palps, individual glossa, fore-tarsi, mid-tarsi and hind-tarsi). We make the assumption that mRNA expression is proportional to protein expression. Validation of this assumption requires antibodies to the honeybee Grs, which are not currently available.

## Materials and methods

### Insects

Forager (≈3 weeks old) worker honeybees (*Apis mellifera* Buckfast strain) were collected returning to one hive situated outdoors at Newcastle University, Newcastle upon Tyne (UK) between July and September, 2013. Bees with pollen on their corbiculae were avoided and all foragers were assumed to be a mix of water and nectar collectors or bees returning with an empty crop. Newly emerged bees (≈24 hours old) were collected from two brood frames taken from the outdoor hive and stored in a mesh box (275 mm X 440 mm X 140 mm) in an incubator at 34°C. Honeybees were captured individually in plastic vials and placed on ice for cold anaesthetisation. When bees ceased moving they were either dissected under a light microscope for immediate measurement of Gr gene expression (forager and newly emerged bees), or approximately 20 individuals (foragers only) were transferred to feeding cages for experimental feeding assays (see below).

### Preparation of brain tissue of starved bees

Following cold anaesthetisation, approximately 60 forager honeybees were restrained in a modified pipette tip using duct tape as described in [40]. Subjects were left to acclimatise at room temperature for 20 min then fed 10 μl of 0.7 M sucrose using a Gilmont syringe (Gilmont Instruments). Following feeding, bees remained at RT without food for 24 h in a humidified box. After 24 h the bees still alive were cooled on ice and the whole brain was dissected directly into TRIzol solution (see below). These brain samples represented the ‘starved’ condition.

### Preparation of brain tissue from bees fed specific sugars

Following cold anaesthetisation forager honeybees were immediately placed in six plastic boxes (approximately 20–30 bees per box, 11W x 20L x 6H cm) and allowed to recover at RT for approximately 1 h without access to food. Experimental solutions were added and boxes were placed in a temperature controlled room 34 ± 1°C, 60 ± 5% relative humidity (RH) and kept under a D:L 22h:2h light regime. The darkness was intended to replicate the interior of the hive and the 2h light occurred during the period of the day in which the experimental diets were being changed.

Feeding solutions were provided via 2 ml microcentrifuge tubes inserted horizontally. One of three carbohydrate solutions was provided to the bees over a 96 h period: 0.7M sucrose, 0.7 M fructose or 0.7 M glucose. One water tube remained available to bees at all times. Tubes were replaced by a new tube containing a fresh solution every 24 h.

### Extraction and reverse transcription of RNA

For 'hard' tissues, 75 honeybees were dissected and parts were pooled into biological samples containing 75 individual tissues (1 sample for glossa and 2 for duplicated appendages e.g. tarsi, antennae, labial palps, galea). Smaller numbers of individual body parts did not yield sufficient mRNA to measure Gr expression. Body parts collected were: both antennae, both galea, both labial palps, individual glossa, 6 tarsi separated into pairs: fore-tarsi, mid-tarsi and hind-tarsi. Tarsi consisted of five tarsomeres, including basitarsus, distal pretarsus and tarsal claw. Dissected body parts were immediately transferred into 500 μl of TRIzol reagent (Invitrogen) and to -80°C storage until homogenisation.

The RNA yield from 'soft' tissues (>0.4μg/mg of tissue) allowed the use of pooled samples from fewer whole brains and guts, 20 in total (4 biological replicates consisting of 5 pooled individual tissues, guts: from the crop to the rectum). These were immediately transferred into 1 ml of TRIzol reagent and placed in -80°C until further processing.

Separate ‘hard’ and ‘soft’ tissue samples were collected from both forager and newly emerged honeybees.

All hard tissue samples were removed from -80°C freezer, allowed to thaw and homogenised by hand using an Eppendorf micropestle (Sigma-Aldrich), a further 500 μl of TRIzol was added to each sample (1 ml total). Soft tissues did not require homogenisation.

Total RNA was extracted using TRIzol reagent (Invitrogen) with a few modifications to the manufacturer’s protocol that were necessary to achieve sufficient RNA to quantify Grs expressed at low levels. Chloroform (200 μl) was added to each sample, followed by vigorous shaking (15 s) then incubation (3 min at RT) and centrifugation (15 min at 12,000g, 4°C). The aqueous phase was removed and added back to 750 μl of TRIzol reagent. The purification procedure was repeated and the aqueous phase was transferred to a clean microcentrifuge tube. Isopropanol (500 μl, Sigma-Aldrich) and co-precipitant (2.5 μl Glycoblue, Ambion) was added to each sample and samples were held at -80°C overnight (min. 12 h). RNA was collected by centrifugation (10 min at 12,000g) then pellets were washed twice with 75% ethanol. RNA was allowed to air dry then suspended in 20 μl of RNase/DNase-free water. Samples were treated with RNase-free DNase (Promega) following the manufacturer’s instructions then RNA yield was determined using a Nanodrop spectrophotometer ND-1000. Optical density ratios for all samples were >1.8 for both 260/280 and 260/230. Reverse transcription was carried out using Superscript III reverse transcriptase (Invitrogen), following the manufacturer’s instructions.

### Polymerase chain reaction

Standard PCR was carried out using MyTaq HS Mix DNA polymerase (Bioline) to confirm specificity of primers. Thermal cycling parameters after denaturing at 95°C for 1 min were: 35 cycles of 95°C for 15 s, 55°C (for Gr genes) or 60°C (for reference genes) for 15s, 72°C for 10 s. PCR products were sequenced by Geneius Labs (Cramlington, UK). Primers were manually designed (S1 Table).

Quantitative real time-PCR was performed on a Roche LightCycler 480 using LightCycler SYBR Green I Master (Roche), 0.25 μM of each primer and 1 μl cDNA. Each sample was run in duplicate with the following cycling parameters: 95°C for 5 min, 50 cycles of 95°C for 15 s, 55°C for 30 s, 72°C for 1 min, followed by one cycle of 95°C for 5 s and 65°C for 1 min, then 40°C for 10 s.

Relative mRNA expression was calculated using the 2ΔΔCt method [41] against the reference gene RP49 (RPL32, Gene ID: 406099, [42]) and relative to the level in forager brain for which the expression of gustatory receptor 1 (Gr1) was assigned a value of 1. A standard curve was run for each gene in order to confirm amplification efficiencies for each primer set, including the reference gene. We compared expression between different genes by running reactions with all primer sets on a pooled brain tissue sample on a single plate, then included this sample as the standard against which all other tissues were normalised. Samples were additionally analysed against a second reference gene, RPS8 (Gene ID: 406126, [16]), as a control for differences in reference gene expression between age groups (S2 and S3 Figs).

### Statistical analyses

Each external tissue sample was 75 body parts that were pooled and analysed as one or two biological replicates therefore no statistical analyses were carried out on expression levels of any Gr in any tissue except brains and guts (four biological replicates each consisting of five pooled body parts). Measurements of gustatory receptor mRNA in brain and gut samples were analysed using SPSS version 21.0. A generalised linear model (GZLM) was carried out separately for each gustatory receptor with age (forager vs newly emerged) and body part (brain vs gut) used as independent variables. A pairwise comparison was carried out with Sidak adjustment for multiple comparisons.

In the feeding manipulations (both the fed versus starved conditions and the carbohydrate diets) expression of the candidate sugar receptor genes (AmGr1, AmGr2 and AmGr3) in the brain of forager bees were analysed using a One-Sample Wilcoxon Signed Rank Test. The one sample test was carried out on each Gr individually, comparing each sample to the appropriate control which was always normalised to 1.0. All gene expression was compared between the same gene in the 0.7 M sucrose *ad libitum* 96 h feeding ‘control’ condition which was normalised to 1.0 in all tests respectively.

## Results

### Gustatory receptor gene expression is widespread and is greater in the taste organs than in internal tissues

**Fig 1:**
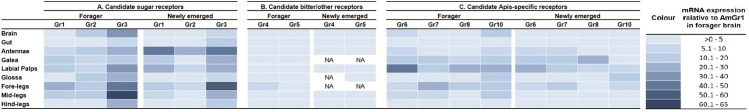
Gustatory receptor mRNA levels are not equal in every body part in both newly-emerged and forager honeybees. All expression levels are relative to the expression of the reference gene RP49 (RPL32) and are normalised to AmGr1 in the forager brain. **A.** Expression levels of the candidate sugar receptors (AmGr1 and AmGr2) and fructose receptor (AmGr3) genes across the un-manipulated forager (≈2–3 wk old) and newly emerged honeybee (≈24 h old) anatomy. **B.** Expression levels of the candidate bitter receptors in forager and newly emerged bees (NA represents unavailable data.). **C.** Expression levels of the unknown and potentially *Apis*-specific receptor genes in forager and newly-emerged bees. Note: AmGr9 mRNA expression levels were detected in all tissue types in both groups however levels were too low to include reliable expression values. Cells are shaded to indicate level of expression. N^soft tissue^: 20 individual tissues. N^hard tissue^: 75–150 individual tissues.

Expression of mRNA for all 10 honeybee Grs was observed in all tested tissue types in both forager and newly emerged bees (but Gr9 mRNA was at levels too low to quantify reliably) (Fig 1). The candidate bitter receptor genes, Gr4 and Gr5, had the lowest expression levels of any of the 9 genes we could quantify, and expression of these was relatively stable across all tissue types in both age groups (Fig 1B). Overall, expression of the other Gr genes was higher in the taste organs than in the internal tissues (gut and brain) (Fig 1A and 1C).

Of the three genes thought to encode receptors for sugar detection, the candidate fructose-sensing gene (Gr3) was expressed at greatest levels across all forager tissues and the majority of tissues of newly emerged bees. The two candidate sugar-sensing genes (Gr1 and Gr2) were expressed at lower levels than Gr3 in the brain, labial palps and all tarsi (Fig 1A). The mRNA levels of these three Gr genes in the antennae of newly-emerged bees were almost double that of the antennae of foragers. The expression of Gr3 mRNA was also higher in the fore-tarsi of newly emerged bees (Fig 1A).

For the remaining four candidate ‘*Apis*-specific’ genes that could be quantified, the expression levels were more variable. Internal expression (brains and guts) of these four was notably higher in the foragers compared with newly emerged bees (Fig 1C), with Gr6 and Gr10 generally showing greater expression levels than Gr7 or Gr8. For both age groups the highest levels of expression for all these four genes is generally in the mouthparts, primarily the labial palps and the galea, with Gr6 dominating (Fig 1C). Additionally, the Gr7 gene is expressed at the greatest level in the fore-tarsi of both age-groups.

### Expression in internal tissues is greatest in the forager brain

All 10 Gr mRNAs were detected in both the brain and gut of newly emerged and forager honeybees (Fig 1, S1 Fig). The mRNA for all but one Gr gene (Gr8) was greater in the forager brain compared with the forager gut. Whereas individual Gr mRNA levels in the guts of the two age groups was similar (Fig 1, Table 1, S1 Fig), in brain most mRNAs were higher in the foragers than in newly-emerged adults.

**Table 1:** GZLM for gustatory receptor expression in brains and guts of newly emerged and forager honeybees with age and body part as independent variables for a full factorial analysis.

	Age			Internal body part			Age*Internal body part		
	x^2^	df	P-value	x^2^	df	P-value	x^2^	df	P-value
**Gr1**	8.3	**1**	**0.040**	2.0	**1**	**0.159**	10.0	**1**	**0.020**
**Gr2**	5.3	**1**	**0.022**	4.3	**1**	**0.039**	3.6	**1**	0.059
**Gr3**	25.0	**1**	**<0.001**	22.3	**1**	**<0.001**	41.9	**1**	**<0.001**
**Gr4**	30.6	**1**	**<0.001**	18.1	**1**	**<0.001**	20.4	**1**	**<0.001**
**Gr5**	98.5	**1**	**<0.001**	115.3	**1**	**<0.001**	93.4	**1**	**<0.001**
**Gr6**	10.8	**1**	**0.001**	25.5	**1**	**<0.001**	14.	**1**	**<0.001**
**Gr7**	33.3	**1**	**<0.001**	24.0	**1**	**0.001**	23.5	**1**	**0.001**
**Gr8**	5.6	**1**	**0.018**	2.1	**1**	0.152	1.8	**1**	0.186
**Gr10**	42.7	**1**	**<0.001**	31.0	**1**	**<0.001**	35.7	**1**	**<0.001**

All *P*-values < 0.05 are shown in bold. N = 4 Pooled biological replicates consisting of 5 brains or 5 guts each (20 bees total).

### Expression of the candidate sugar receptors in the forager brain is dependent on nutritional status and energy source

We proposed that the relatively high levels of expression of some Grs in the forager brain was a result of regulation in relation to satiety state and/or specific source. Testing this hypothesis with respect to specific Grs requires knowledge of their ligand, and functional analysis of the honeybee Grs is lacking. However, due to relatively strong orthology with *Drosophila* sugar receptors [16, 18] and a single study on the sugar ligands of AmGr1 and AmGr2 [19] we reasoned that ligands could be assigned with reasonable confidence to the three candidate sugar receptor genes (*AmGr1*, *AmGr2* and *AmGr3*).

**Fig 2:**
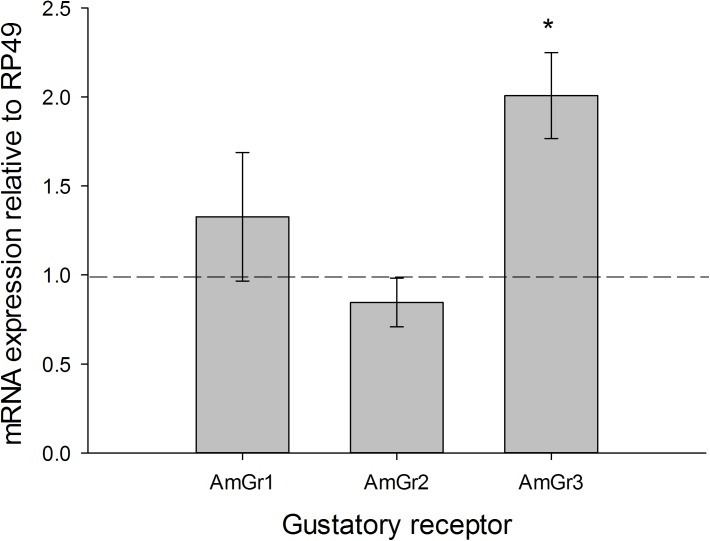
mRNA expression of the three candidate sugar receptors, AmGr1, AmGr2 and AmGr3 in the brains of ‘starved’ forager honeybees (provided with 10μl of 0.7M sucrose then held for 24 h without food). All expression levels are relative to mRNA expression of the reference gene RP49 (RPL32) in the brain. The expression of each gene has been normalised to the mRNA expression of that same gene under 0.7M sucrose a*d libitum* feeding conditions over 96h, ‘fed’ condition, set at a value of 1.0 (represented by the hashed line). Expression levels are not comparable between genes. *: P < 0.05 One-Sample Wilcoxon Signed Rank test. N = 3–4 biological replicates (15–20 whole brains measured as mRNA pooled from groups of 5 brains).

To test if expression of these candidate sugar receptors is determined by satiety state we compared the concentration of mRNA in the brains of forager honeybees that had been fed only a small volume of 0.7 M sucrose solution (10μl) and starved for 24 h with the concentration in brains of bees that had *ad libitum* access to 0.7 M sucrose solution for 96 h. AmGr3 mRNA was elevated approximately 2-fold in the brains of starved foragers (Fig 2) compared with the *ad libitum* fed foragers (One-Sample Wilcoxon Sign Rank Test, P = 0.012). Expression of AmGr1 (One-Sample Wilcoxon Sign Rank Test, P = 0.916) or AmGr2 (One-Sample Wilcoxon Sign Rank Test, P = 0.401) was not affected by this feeding regime. Thus, out of the three candidate sugar and fructose receptor genes only AmGr3 expression in forager brain appears to be influenced when bees are starved.

**Fig 3:**
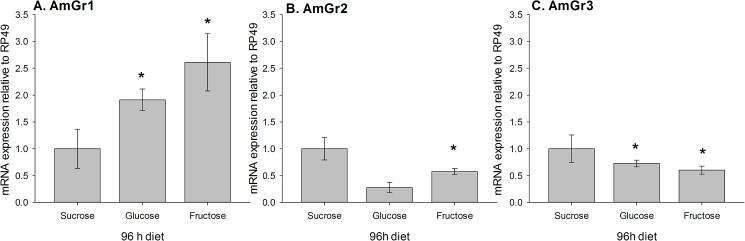
**mRNA expression of the three candidate sugar receptors, A. *AmGr1*, B. *AmGr2* and C. *AmGr3* in the brains of forager honeybees following 96 h *ad libitum* feeding on one of three 0.7 M carbohydrate diets (disaccharide sucrose or either monosaccharide glucose or fructose).** All expression levels are relative to mRNA expression of the reference gene RP49 (RPL32) in the brain. The expression of each gene has been normalised to the mRNA expression of that same gene under 0.7M sucrose *ad libitum* (first bar in each graph), set at a value of 1.0. Expression levels are not comparable between genes. *: P < 0.05 One-Sample Wilcoxon Signed Rank test. N = 2–4 biological replicates (10–20 whole brains, measured as mRNA pooled from groups of 5 brains).

To test if expression of these candidate sugar receptors reflects exposure to specific sugar ligands we measured AmGr1, AmGr2 and AmGr3 mRNA after *ad libitum* feeding with sucrose or the constituent monosaccharides (glucose or fructose) over 96 h. Compared with sucrose, forager-age bees fed with glucose or fructose had greater expression of AmGr1 mRNA in the brain (Fig 3A). The brains of bees fed with 0.7 M glucose diet exhibited a 2-fold increase in expression of AmGr1 mRNA compared to 0.7 M sucrose diet (One-Sample Wilcoxon Sign Rank Test, P = 0.028) and a 2.5-fold increase of AmGr1 mRNA following a 0.7 M fructose diet (One-Sample Wilcoxon Sign Rank Test, P = 0.017). Conversely, both monosaccharide diets resulted in lower mRNA expression for both AmGr2 (Fig 2B) and AmGr3 (Fig 2C). The brains of bees fed with the fructose diet exhibited lower expression of AmGr2 (One-Sample Wilcoxon Sign Rank Test, P = 0.028) and AmGr3 mRNA (One-Sample Wilcoxon Sign Rank Test, P = 0.028). Whereas, bees fed with the glucose only diet exhibited lower levels of AmGr3 mRNA in the brain (One-Sample Wilcoxon Sign Rank Test, P = 0.012). Thus, expression of these Gr genes in forager brain reflects dietary energy source.

## Discussion

Our experiments show that while honeybees possess seemingly few Gr genes (12 Gr genes total) compared with other insects [16, 18], the expression of 10 of these genes is widespread across the honeybee anatomy. Messenger RNA corresponding to all 10 Gr genes was detected in every tissue analysed, including the non-canonical gustatory tissues: brain and gut. In general, however, Gr mRNA levels were low, which made quantification especially challenging. This was the case particularly for Gr9, which we were unable to quantify via RT-qPCR but detected by standard RT-PCR in all tissues analysed.

The primary difference in Gr gene expression between bees in the two age groups measured was the relatively high expression of genes in the forager brains compared with brains of newly-emerged bees. This Gr expression in the forager brain is surprising, and adds to the known series of physiological and hormonal changes that accompany the transition from hive to foraging [21, 43–45]. This finding could indicate that adult forager honeybees sense nutrients internally, an idea that was substantiated by the observation that expression of Grs in forager brain was affected by nutritional status and specific dietary sugar source. Unexpectedly, all quantifiable genes were also discovered in the gut of both newly emerged and forager bees. Expression of Grs in gut has been previously observed in some insect species. However, this is the first example of almost an entire Gr gene repertoire being expressed in the gut. Foraging honeybees have a high metabolic rate to maintain flight and the relatively high concentration of sugars and quantity of nectar they collect allows them to fuel this rapid metabolism [46, 47]. Multiple internal Grs may allow the bee to assess its internal nutritional state more effectively, by detecting different sugars in the gut and/or haemolymph and therefore regulate energy levels through carbohydrate consumption, in order to maintain foraging. In the current work we measured Gr mRNA in the entire gut and the crop. Thus, the specific location of Gr gene expression in the gut remains unknown. Expression in the gut lumen would detect food concentration and may regulate consumption and digestion, whereas expression in the crop may detect recently consumed food and aid movement through the proventriculus. Additionally, Grs may be expressed in the epithelial cells or on the haemolymph side of the gut to assess the concentration of haemolymph nutrients. It is important to note that the same gene may be expressed differentially in different tissues within the same structure [48]

The collection and consumption of carbohydrates is vital to the honeybee. Floral nectar, honey and even honeybee haemolymph often contain high concentrations of carbohydrates [46, 49, 50]. The candidate sugar (Gr1 and Gr2) and candidate fructose (Gr3) receptor genes generally exhibited the highest expression levels of all the 10 Gr genes in all tissues of both forager and newly emerged bees. The strong, widespread expression of candidate sugar receptor genes is probably linked to the high carbohydrate diet of the honeybee. Recently, Jung et al [19] quantified the expression of Gr1 and Gr2 in the honeybee antennal tip and assessed sensitivity using electrophysiological tip recordings. By expressing these receptors in *Xenopus* oocytes, they found that Gr1 responded to sucrose, glucose, trehalose and maltose but not fructose. Interestingly, the sensitivity toward these four sugars was increased when Gr1 and Gr2 were co-expressed [19], indicating a potential heterodimeric complex, similarly to some Grs observed in *Drosophila* [51, 52]. In addition to increasing sensitivity, Gr dimerization in *Drosophila* also increases the range of detectable nutrients [51–54]. The comprehensive Gr expression observed in the internal tissues of the honeybee may be related to function as heterodimers. The ability to detect nutrients internally in a manner that is comprehensive, flexible and sensitive would facilitate advantageous adaptation to the rapidly changing haemolymph titres of the honeybee. Therefore, internal Gr expression may be contributing to sugar homeostasis in the honeybee.

Internally expressed Grs acting as nutrient sensors were identified initially in mammals. In the rodent gut, the sodium-dependent glucose transporter isoform 1 (SGLT1) protein is responsible for transporting dietary sugars from the intestinal lumen into enterocytes. Taste receptors from the T1R family, along with the G-protein gustducin, were also found in the gut and play a role in carbohydrate sensing and absorption by regulating expression of SGLT1 mRNA and protein [55, 56]. The internal expression of Grs in insects however, has only recently been reported [9–11]. For example, the first study on Gr expression in the *Drosophila* midgut discovered 12 out of the existing 68 Grs in enteroendocrine cells that were co-localised with three regulatory peptides (neuropeptide F, NPF; locusta tachykinin, LTK and diuretic hormone 31, DH31), suggesting a role in food uptake and nutrient regulation [10]. However, no specific function was identified and only one study to date has provided direct evidence for a Gr functioning as a nutrient sensor in *Drosophila* [11].

We suggest that the increase in AmGr3 we observed in starved bees and the reduction we observed in bees fed fructose is a component of the circuitry that senses and regulates the intake of this sugar. Sucrose is a disaccharide of glucose and fructose and additionally glucose can be converted to fructose via the polyol pathway [57]. Therefore, fructose sensing may be an accurate way to detect low levels of all three tested nutrients. The increase in (fructose-sensing) AmGr3 in starved bees may reflect a ‘scavenging response’ to drive consumption of this nutrient when alternative energy sources are scarce (as signalled by low haemolymph glucose and fructose concentration). A study in *Drosophila* provides a precedent for this idea. Starved flies expressed more DmGr64a and had a lower threshold for sucrose detection, which was interpreted as higher gene expression leading to greater sensitivity to sucrose to afford starvation-resistance to hungry flies via the acceptance of less nutritious food components [58]. Following the ‘increased expression equates to increased sensitivity’ hypothesis we could posit that AmGr1 is primarily a candidate for sucrose detection, therefore expression and subsequently sensitivity increases when bees receive a sucrose-free diet. However, considering the small quantity of sucrose likely to reach the haemolymph un-metabolised, this is unlikely. Alternatively, there are a whole host of sugars that bees respond to [2, 59], and this receptor could primarily be responsible for the detection of one or more of those compounds. However, we must also note that the differential expression of Gr genes in the honeybee brain as a function of sugar consumption does not confirm that these receptors function as gustatory receptors in the brain. Changes in expression level may be resulting from a nutrient sensing pathway that doesn’t involve the Gr genes directly.

Unexpectedly, gustatory receptor mRNA expression levels in the antennae of newly-emerged bees were almost double the expression in the antennae of foragers, especially for the candidate sugar receptors (Gr1, Gr2 and Gr3). However, as all the antennal analysis was carried out on pooled samples it is difficult to determine if this is the case for all newly emerged bees. Adult honeybees undergo task differentiation as they age within the hive. Shortly after emergence, young adult workers care for brood and the queen including the production of royal jelly and regulating temperatures around the brood [60]. The completion of such tasks is strongly mediated by chemical and pheromone signals that the bees interpret through antennal assessment. An enhanced level of Gr expression on newly-emerged bees’ antennae may promote efficient assessment of the brood, particularly for food presence in the larval cells and larval feeding status.

In contrast to the current work, the original study by Robertson and Wanner [16] found the candidate sugar and fructose receptor genes had the lowest expression levels within four honeybee tissues. It is difficult to compare their work as our study used the brain as the reference tissue for Gr expression, whereas [16] used the honeybee body (without heads and legs). Importantly, they did not provide information about comparisons between level of expression of each gene. In our study, the expression of all Grs were arbitrarily referenced to the expression of Gr1 in the brain.

In the honeybee there are only two additional genes that maintain any homology with Grs in other insects, although the sequence similarity is considerably weaker than those of the sugar and fructose receptors. The Gr genes Gr4 and Gr5 are most similar to the conserved *Drosophila* complex DmGr28a-e [16]. This complex has been identified in labellar bitter neurons [61] and the high level of conservation in this complex lends support to the honeybee receptors mediating a similar function. In *Drosophila*, Gr28b.d functions as a thermosensor [62]. In the honeybee, thermosensing has been mainly attributed to a Transient Receptor Potential A channel (AmHsTRPA). Interestingly this receptor also functions as a chemosensor for molecules such as camphor [63] and so it is possible that Gr4 or Gr5 could be co-receptors with other Gr genes, or also be used in other, non-gustatory functions including thermosensing, but this has not yet been tested. The Gr4 and Gr5 genes have also been highlighted as a species-specific duplication as only one similar gene has been identified in the bumblebee, *Bombus terrestris* (BtGr4PSE), and this is now considered a pseudogene [18].

The remaining gustatory receptor genes (Gr6, Gr7, Gr8, Gr9 and Gr10), were expressed at the highest levels (excluding Gr9) in the mouthparts. Of all the taste organs, the mouthparts are the only areas thus far to have been identified in electrophysiological studies to have neurons that spike in response to stimulation with toxic compounds [64, 65, 67]. The fact that these Grs are expressed at the highest level in the mouthparts may indicate one or more of these Grs function as a receptor for the detection of bitter compounds. The corresponding genes in *B*. *terrestris* show enhanced duplication and the potential to detect bitter compounds has been reported [18]. These Grs could also permit the detection of nutrients, such as amino acids or carbohydrates. For example, hummingbirds lack the vertebrate sweet taste receptor T1R2 [66] but the structure of the heterodimeric mammalian umami receptor, T1R1-T1R3, appears to have adapted in this species such that it permits carbohydrate detection [66]. Similarly, the high carbohydrate diet of the honeybee may have driven an evolutionary adaptation of receptor function to promote sugar detection.

While the number of Gr genes possessed by honeybees seems low it is important to note that gustatory receptors may not solely be responsible for the general perception of taste. Work on insect ionotropic receptors (IRs) is gaining momentum, and recently a number of studies have highlighted that IRs play a role in gustation [68–70]. To date honeybees are thought to possess 21 IRs [18], some or all of which may act independently or in conjunction with gustatory receptors to increase the scope or sensitivity of the honeybee gustatory system. However, further detailed analysis to identify the ligands of the 10 *Apis* Grs is required to gain an understanding of this system and its potential interaction with other molecules.

## Supporting information

S1 Table Apis mellifera gustatory receptor gene information and associated primers. (DOCX)

S1 Fig Expression levels in internal tissues of most gustatory receptor mRNA is greatest in the forager honeybee brain. Expression of mRNA for *Apis mellifera* gustatory receptors (AmGr): in newly-emerged and forager bees, brain and gut tissues **A.** AmGr1 (N = 3–4 biological replicates), **B.** AmGr2 (N = 3–4 biological replicates), **C.** AmGr3 (N = 4 biological replicates), **D.** AmGr4 (N = 3–4 biological replicates), **E.** AmGr5 (N = 2–4 biological replicates), **F.** AmGr6 (N = 3–4 biological replicates), **G.** AmGr7 (N = 3–4 biological replicates), **H.** AmGr8 (N = 2–4 biological replicates), **I.** AmGr10 (N = 3–4 biological replicates). Data are mean ± SEM. All mRNA levels are relative to the reference gene RP49 and values are all normalised to the level of expression in the forager brain. a, b, c represent GZLM pairwise comparison, Sidak *P* < 0.05.
(TIF)

S2 Fig Raw Ct values from the RT-qPCR for the two reference genes Ribosomal Protein 49 (RP49) and Ribosomal protein S8 (RPS8) showing relatively stable expression across all assessed body parts of forager (~2–3 weeks old) and newly emerged (~24 h old) honeybees. Body parts: Brain (N = 20 pooled tissues), Gut (20 pooled tissues), Ant: antenna (N = 150 pooled tissues), Galea (N = 150 pooled tissues), Glos: Glossa (N = 75 pooled tissues), L palps: Labial palps (N = 150 pooled tissues), F-tarsi: fore-tarsi (150 pooled tissues), M-tarsi: Mid-tarsi (150 pooled tissues), H-tarsi: Hind-tarsi(150 pooled tissues).
(TIF)

S3 Fig Expression levels of the 10 honeybee gustatory receptor genes across the un-manipulated forager (≈2–3 wk old) and newly emerged honeybee (≈24 h old) anatomy, as seen in Table 1, relative to RPS8 as a reference gene (NA represents unavailable data). (TIF)
