# Supplementary material for: Effects of age and nutritional state on the expression of gustatory receptors in the honeybee (Apis mellifera)
Source: PLoS One. 2017 Apr 12;12(4):e0175158. doi: 10.1371/journal.pone.0175158 (PMC5389653; doi:10.1371/journal.pone.0175158)
Supplement: S1 Table — (DOCX) [file pone.0175158.s001.docx]

| **Gustatory receptor/ Ribosomal protein** | **WGS accession number** | **Length of amplicon** | **GC content** | **Primer length** | **Tm** | **Primers designed 5'-3'** | **Location in whole genome shotgun sequence** | **Annealing Temerature for RT-PCR (^o^C)** |
| --- | --- | --- | --- | --- | --- | --- | --- | --- |
| **AmGr1** | NC_007074.3 | 358 | 40%  45% | 20  20 | 53.2  55.3 | Forward primer:  ATCGATAATCCACGGTTACT  Reverse primer:  CAGTTGTCTCGTTAAGGTTG | Amel_4.5 gi\|323388983  Range FOR: 1: 10441164 to 10441144. Range REV: 1: 10440476 to 10440456. | 55 |
| **AmGr2** | NC_007074.3 | 108 | 47%  47% | 19  19 | 54.5  54.5 | Forward primer: CGCTCAAATATTCGGCATG  Reverse primer:  GGCGATGAAACCTGAATAC | Amel_4.5 gi\|323388983 Range FOR 1: 13083333 to 13083352. Range REV: 1: 13083244 to 13083225. | 55 |
| **AmGr3** | NC_007077 | 171 | 33%  48% | 21  21 | 52  57.9 | Forward primer:  GCGTACTTGTATTACTACTTA  Reverse primer:  GGAAAGGAGAGCCAACAATAC | Amel_4.5 >gi\|323388980 Range FOR: 1: 2916885 to 2916864 Range REV: 1: 2916735 to 2916714. | 55 |
| **AmGr4** | NC_007082.3 | 168 | 47%  50% | 17  18 | 50.4  53.7 | Forward primer:  CATCGTTTGCAACAACC  Reverse primer:  GCCTGCGAAAATTGTAGG | Amel_4.5 >gi\|323388975  Range FOR: 1: 588751 to 588768. Range REV: 1: 588901 to 588919. | 55 |
| **AmGr5** | NC_007070.3 | 217 | 50%  50% | 20  18 | 57.3  53.7 | Forward primer:  GTACGATCGATCGAGAAACG  Reverse primer:  CTGCATTGCGTGCAATTG | Amel_4.5 >gi\|323388987  Range FOR: 1 21211577 to 21211597. Range REV: 1: 21211928 to 21211946. | 55 |
| **AmGr6** | NC_007072.3 | 209 | 44%  50% | 18  18 | 51.4  53.7 | Forward primer:  CAGATGAATGTTTCCGTG  Reverse primer:  CGAATACAAGAGCGAGTC | Amel_4.5 >gi\|323388985  Range FOR: 1: 964294 to 964276. Range REV: 1: 964103 to 964121. | 55 |
| **AmGr7** | NC_007078.3 | 190 | 44%  47% | 18  19 | 51.4  54.5 | Forward Primer:  GGCAACATTATTTGCGAG  Reverse primer:  CTTGGATCATGACTACGAG | Amel_4.5 >gi\|323388979  . Range FOR 1: 10261600 to 10211582. Range REV: 1: 10261285 to 10261266 | 55 |
| **AmGr8** | NC_007082.3 | 277 | 40%  58% | 20  19 | 53.2  58.8 | Forward primer:  CAATACAGAAGTAGGCAAGA  Reverse primer:  GCACGTCATGTCCGTCACA | Amel_4.5 >gi\|32338897  Range FOR: 1: 5972720 to 5972740. Range REV: 1: 5973043 to 5973062 | 50 |
| **AmGr9** | NC_007082.3 | 288 | 35%  50% | 23  20 | 55.3  57.3 | Forward primer:  GCATTTAGAGGAGAAACATTTAG  Reverse primer:  GCGTCATAAAGGGTCCACTT | Amel_4.5 >gi\|323388975  Range FOR: 1: 5974245 to 5974268. Range REV: 1: 5974513 to 5974533. | 53 |
| **AmGr10** | NC_007073.3 | 293 | 45%  59% | 20  17 | 55.3  55.2 | Forward primer:  CTGACAAGATAATAGAGGCG  Reverse primer:  ATTCGCCTGATGAGCCG | Amel_4.5 >gi\|323388984  Range FOR: 1: 11145805 to 111455785. Range REV: 1: 11145529 to 11145431  . | 55 |
| **RP49** | AF441189.1 | 100 | 41%  30% | 22  27 | 56.5  57.4 | Forward primer: GGGACAATATTTGATGCCCAAT  Reverse primer: CTTGACATTATGTACCAAAACTTTTCT | Amel_4.5 >gi\|18479060  Range FOR: 1: 945031 to 945053. Range REV: 1: 945103 to 945130. | 60 |
| **RPS8** | NM_001011604.3 | 182 | 55%  65% | 20  20 | 59.4  63.5 | Forward Primer: GGTGCGAAACTGACTGAAGC  Reverse primer: TCCTCACGACCGCACTGTCC | Amel_4.5 >gi\|323388979  : Range FOR 6172001 to 6171981. Range REV: 6171486 to 6171466. | 60 |
