# Supplementary figures and images for: Effects of age and nutritional state on the expression of gustatory receptors in the honeybee (Apis mellifera)
Source: PLoS One. 2017 Apr 12;12(4):e0175158. doi: 10.1371/journal.pone.0175158 (PMC5389653; doi:10.1371/journal.pone.0175158)

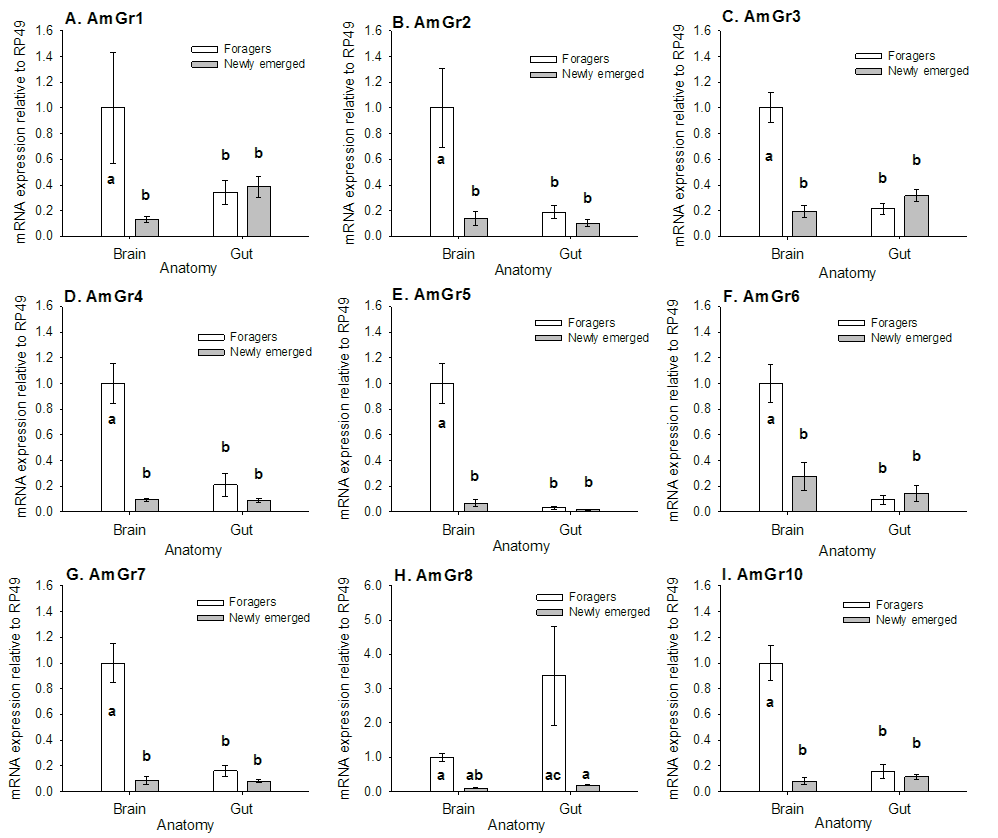

Supplement: S1 Fig — Expression of mRNA for Apis mellifera gustatory receptors (AmGr): in newly-emerged and forager bees, brain and gut tissues A. AmGr1 (N = 3–4 biological replicates), B. AmGr2 (N = 3–4 biological replicates), C. AmGr3 (N = 4 biological replicates), D. AmGr4 (N = 3–4 biological replicates), E. AmGr5 (N = 2–4 biological replicates), F. AmGr6 (N = 3–4 biological replicates), G. AmGr7 (N = 3–4 biological replicates), H. AmGr8 (N = 2–4 biological replicates), I. AmGr10 (N = 3–4 biological replicates). Data are mean ± SEM. All mRNA levels are relative to the reference gene RP49 and values are all normalised to the level of expression in the forager brain. a, b, c represent GZLM pairwise comparison, Sidak P < 0.05. (TIF) [file pone.0175158.s002.TIF]

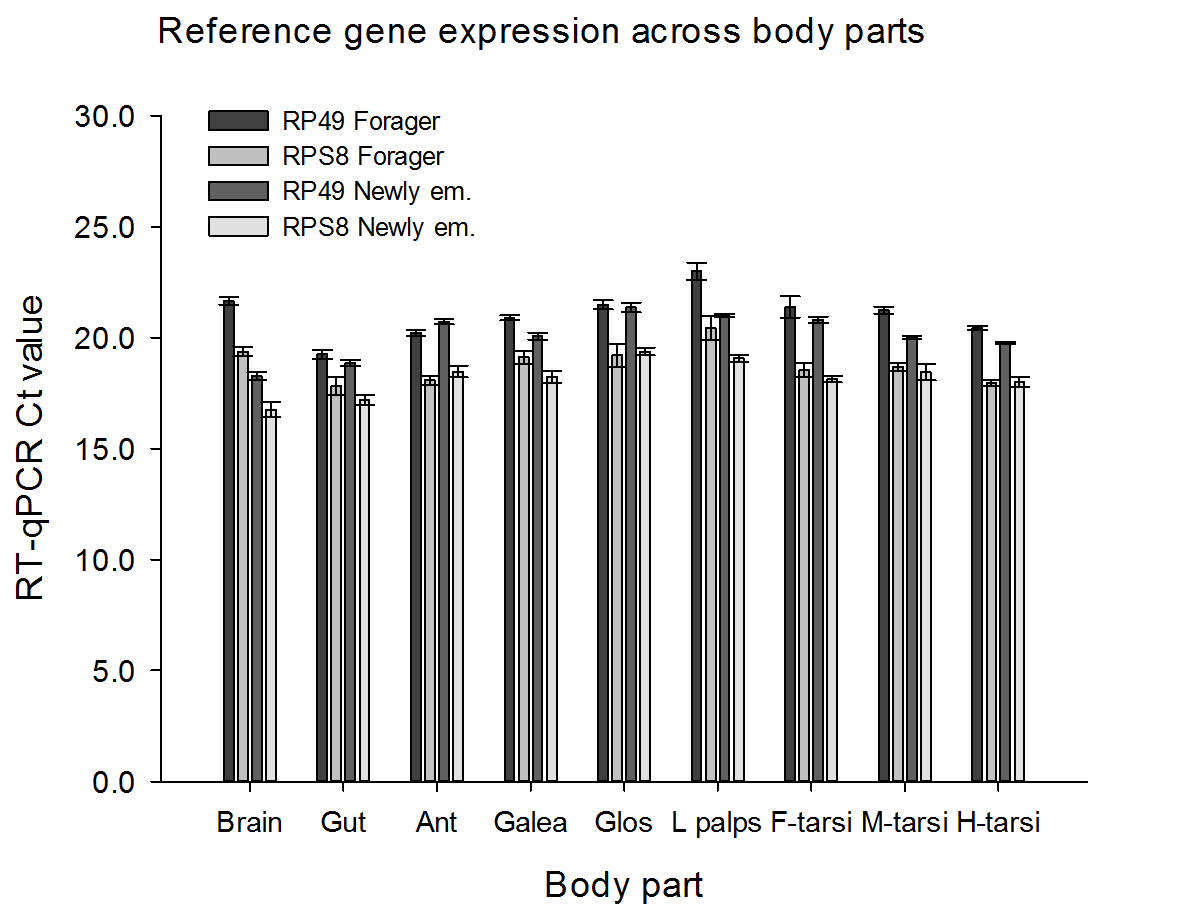

Supplement: S2 Fig — Body parts: Brain (N = 20 pooled tissues), Gut (20 pooled tissues), Ant: antenna (N = 150 pooled tissues), Galea (N = 150 pooled tissues), Glos: Glossa (N = 75 pooled tissues), L palps: Labial palps (N = 150 pooled tissues), F-tarsi: fore-tarsi (150 pooled tissues), M-tarsi: Mid-tarsi (150 pooled tissues), H-tarsi: Hind-tarsi(150 pooled tissues). (TIF) [file pone.0175158.s003.tif]

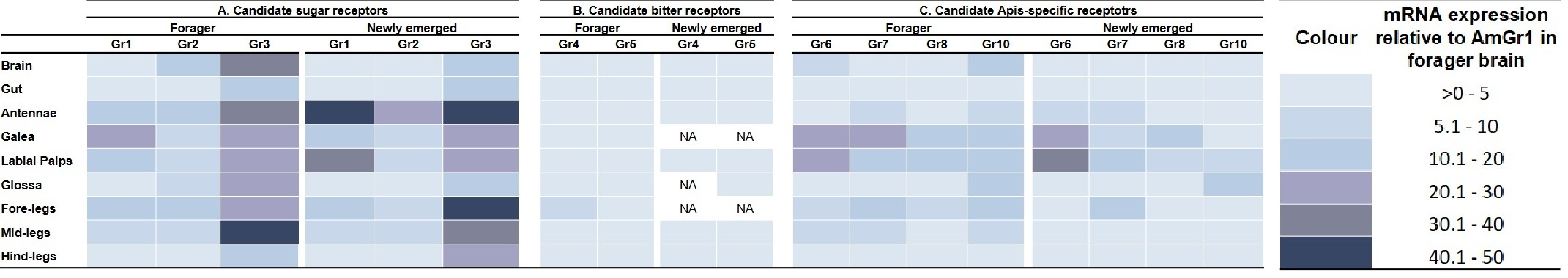

Supplement: S3 Fig — (TIF) [file pone.0175158.s004.tif]
